# Study of Carotid Atherosclerosis in Non-alcoholic Fatty Liver Disease Patients and Its Correlation With Serum Transaminases

**DOI:** 10.7759/cureus.90500

**Published:** 2025-08-19

**Authors:** Akshayaa K Aggarawal, Abhishek Deepak

**Affiliations:** 1 Internal Medicine, Walsall Manor Hospital, Walsall, IMN; 2 Internal Medicine, Shri Ram Murti Smarak Institute of Medical Sciences, Bareilly, IND

**Keywords:** carotid intima-media thickness, dyslipidemia, non-alcoholic fatty liver disease, serum transaminases, subclinical atherosclerosis

## Abstract

Background

Non-alcoholic fatty liver disease (NAFLD) is increasingly recognized as a manifestation of metabolic syndrome and a potential risk factor for cardiovascular disease (CVD). Carotid intima-media thickness (CIMT) is a validated marker of subclinical atherosclerosis which can predict the potential risk factor for CVD. This case-control study aimed to evaluate the association between NAFLD and CIMT and to investigate related metabolic and biochemical parameters.

Objectives

The study aimed to evaluate carotid atherosclerosis in patients with NAFLD by measuring CIMT and to determine its correlation with serum transaminase levels, specifically alanine aminotransferase (ALT) and aspartate aminotransferase (AST).

Methodology

This hospital-based case-control study included 50 patients diagnosed with NAFLD and 50 age- and sex-matched healthy controls. All participants underwent comprehensive clinical assessment, ultrasonographic grading of hepatic steatosis, CIMT evaluation via ultrasound, and laboratory investigations including liver function tests and lipid profiles.

Results

NAFLD was more prevalent in older adults, particularly males, and was closely associated with obesity, diabetes, and dyslipidemia. Most NAFLD cases were classified as mild (Grade I), and a significant number exhibited elevated triglycerides. The CIMT values were significantly increased in NAFLD patients across all age groups, with the most notable rise observed in individuals over 50 years of age (p<0.001). CIMT was positively associated with the severity of fatty liver and showed a significant correlation with ALT (p<0.039), but not with AST levels (p<0.327). A p-value of <0.05 was considered statistically significant.

Conclusion

NAFLD is associated with an increased burden of metabolic dysfunction and early vascular changes as reflected by elevated CIMT. The findings highlight a significant association between ALT levels, triglycerides, and subclinical atherosclerosis, emphasizing the need for routine cardiovascular risk stratification in patients with NAFLD, especially those aged over 50.

## Introduction

Non-alcoholic fatty liver disease (NAFLD) is defined by the accumulation of fat exceeding 5% of liver weight and encompasses a spectrum of hepatic pathology ranging from simple steatosis to non-alcoholic steatohepatitis (NASH), early fibrosis, and cirrhosis, with potential progression to hepatocellular carcinoma (HCC) [1]. In recent years, NAFLD has emerged as the most prevalent chronic liver disease in Western countries among individuals without a history of significant alcohol consumption. In India, epidemiological studies estimate a prevalence of 10-32%, with higher rates observed in overweight individuals and those with diabetes or pre-diabetes [2].

Although NAFLD has traditionally been diagnosed by the exclusion of other causes of chronic liver disease, accumulating evidence has highlighted its strong association with metabolic syndrome and frequent coexistence with other liver pathologies. The European Association for the Study of the Liver (EASL) recognized this in its 2010 position statement, introducing the concept of "primary NAFLD" in its 2016 guidelines, defined as NAFLD associated with metabolic risk factors [3]. In 2020, Eslam et al. proposed the term metabolic dysfunction-associated fatty liver disease (MAFLD), diagnosed in adults with hepatic steatosis (by imaging, biomarkers, or histology) in the presence of overweight/obesity, type 2 diabetes mellitus (T2DM), or at least two metabolic risk abnormalities [4]. Most recently, in June 2023, a multi-society Delphi consensus introduced the nomenclature metabolic dysfunction-associated steatotic liver disease (MASLD), effectively replacing NAFLD. The concepts of MAFLD and MASLD share substantial overlap and are considered more appropriate descriptors of the condition's pathophysiology than the historical NAFLD terminology [5].

The prevalence of NAFLD increases with advancing age, type 2 diabetes, obesity, hypertriglyceridemia, hyperinsulinemia, and systolic hypertension [6], all of which are established risk factors for vascular disease. Non-invasive vascular assessment, such as carotid intima-media thickness (CIMT) measurement, serves as a marker of subclinical atherosclerosis and has been shown to predict future adverse vascular events, including coronary, cerebrovascular, and peripheral arterial disease [7]. Hepatic steatosis, often detected incidentally during abdominal ultrasonography performed for unrelated indications, has been associated in multiple cross-sectional studies with increased CIMT and the presence of carotid plaques, underscoring a potential link between NAFLD and systemic vascular pathology [8].

The pathogenesis of NAFLD is thought to involve the "two hits" hypothesis which was first proposed in 1998. The "first hit" is characterized by the accumulation of triglycerides derived from the esterification of free fatty acid (FFA) and glycerol. The latter "second hit" arises from an imbalance of supply, formation, consumption, and hepatic oxidation and disposal of triglycerides. Fibrosis is the final stage or the "third hit" resulting from an imbalance between the rate of hepatocyte death and hepatocyte regeneration. This results in the activation of hepatic stellate cells thereby progressing to fibrosis. [9].

An elevated alanine aminotransferase (ALT) level is a common laboratory finding in patients with NAFLD [6]. Even ALT levels within the normal reference range have been linked to an increased risk of atherosclerosis and cardiovascular disease (CVD) [10]. This raises the broader question of whether individuals with NAFLD should undergo routine screening for subclinical vascular disease. Current epidemiological evidence indicates that increased CIMT, particularly values ≥1 mm at any age, is significantly associated with elevated risk of major adverse cardiovascular events, including myocardial infarction and stroke [11]. Furthermore, non-invasive assessment of vascular function, such as the evaluation of endothelial dysfunction via flow-mediated dilatation (FMD) of the brachial artery, is emerging as a promising tool for predicting future atherosclerotic events. Recent studies have demonstrated the prognostic value of FMD in forecasting a wide range of cardiovascular outcomes, highlighting its potential role in comprehensive vascular risk stratification [12].

There is limited published data on the association between NAFLD and subclinical atherosclerosis in the general patient population. Therefore, the present study was designed to evaluate the relationship between the severity of fatty liver, liver enzyme levels, dyslipidemia, and their collective impact on atherosclerosis, specifically focusing on carotid atherosclerosis.

## Materials and methods

The study population comprised patients who presented to the Department of Medicine at Shri Ram Murti Medical College in Bareilly, India. A total of 100 participants were enrolled and divided into two groups: 50 patients diagnosed with NAFLD and 50 healthy control subjects. The research protocol adhered to ethical standards.

Study design

This was a cross-sectional observational study.

Inclusion criteria

Inclusion criteria for the study were patients diagnosed with fatty liver as confirmed by abdominal ultrasonography and individuals who were above 18 years of age.

Exclusion criteria

Exclusion criteria for the study included individuals with chronic alcohol intake exceeding 210 grams per week for men and 140 grams per week for women, those who tested positive for hepatitis B surface antigen (HBsAg) or antibodies to hepatitis C virus (anti-HCV), and pregnant individuals. Patients with surgical conditions such as common bile duct (CBD) stones, as well as those with diagnosed disorders like Wilson disease, hemochromatosis, or autoimmune hepatitis, were also excluded. Additionally, individuals who did not provide consent to participate in the study were not included.

Outcomes

In this study, the primary outcome was the mean CIMT in patients with NAFLD compared to age- and sex-matched healthy controls.

Secondary outcomes included the correlation of CIMT with the severity of hepatic steatosis, serum ALT and aspartate aminotransferase (AST) levels, and key metabolic parameters such as triglycerides, high-density lipoprotein (HDL) cholesterol, obesity, diabetes, and hypertension. Additional analyses examined age-stratified differences in CIMT, with particular attention to individuals over 50 years of age.

Study procedure

Written informed consent was obtained from all eligible participants. A thorough medical history, including detailed information on alcohol consumption, and a comprehensive physical examination were conducted and recorded using a standardized proforma.

Data collected included relevant personal history, clinical examination findings, and relevant investigations. These investigations comprised blood tests, such as blood glucose, liver function tests, and lipid profile, as well as radiological studies which included abdominal ultrasonography and carotid artery Doppler studies. 

Data collection

CIMT was measured using a high-resolution Acuson X300 Premium Edition ultrasound system (Siemens Healthineers, Erlangen, Germany) in the Department of Radiology. The B-mode images of the right and left common carotid arteries, carotid bulbs, and internal carotid segments were obtained with subjects in a supine position. CIMT was defined as the distance between the lumen-intima and media-adventitia interfaces of the far wall, with clear echogenic borders enabling both manual and automated measurements.

Abdominal ultrasonography was performed using the Acuson X 700 machine with a 4 MHz electronic probe in the Department of Radiology. Patients diagnosed with fatty liver, characterized by diffuse hepatic echogenicity relative to the renal cortex, were included in the study. 

The severity of fatty liver was classified as non-fatty, mild, moderate, or severe based on ultrasonographic criteria, including increased hepatic echogenicity (bright liver), hepatorenal echo contrast, blurring of intrahepatic vessels, and deep attenuation of the ultrasound signal.

The blood investigations were done in the Department of Hematology and Biochemistry. The blood sugar was measured by the ortho-toluidine method. The liver function test and lipid profile were estimated in an automated clinical chemistry analyzer.

Statistical analysis

Data was analyzed using IBM SPSS Statistics for Windows, V. 23.0 (IBM Corp., Armonk, NY, USA). Data are expressed as mean±standard deviation for continuous variables and as number (percentage) for categorical variables. Differences between cases and controls for continuous variables were assessed using the independent samples Student's t-test. Categorical variables were compared using the chi-squared test. A p-value of <0.05 was considered statistically significant.

## Results

In this study, 50 participants with NAFLD and 50 matched controls were included, bringing the total sample size to 100. A detailed analysis of the key aspects of the study is presented below.

Baseline characteristics of the study population

In the present study, males predominated in both groups, accounting for 64% of NAFLD cases and 52% of controls. The majority of participants were middle-aged, with 72% of NAFLD cases and 70% of controls aged 41 years or older; the 51-60-year age group formed the largest proportion in both groups.

Overweight individuals constituted the highest proportion in both cases (46%) and controls (62%), while obesity (BMI ≥30 kg/m²) was more prevalent among NAFLD cases (32%) than controls (24%).

Smoking prevalence was similar between groups (34% in cases vs. 32% in controls). Diabetes was more common in NAFLD cases (54%) compared to controls (42%), whereas the prevalence of impaired fasting glucose was marginally higher in cases (20%) than in controls (16%). Hypertension was observed in 34% of cases and 40% of controls (Table 1). 

**Table 1:** Baseline characteristics of the study population Data have been represented as n and the percentage of patients. The n is the number of patients.

Characteristic	NAFLD cases (n=50)	Controls (n=50)
Sex	Male: 32 (64%)	Male: 26 (52%)
	Female: 18 (36%)	Female: 24 (48%)
Age group (years)	<40: 7 (14%)	<40: 5 (10%)
	41-50: 7 (14%)	41-50: 10 (20%)
	51-60: 16 (32%)	51-60: 21 (42%)
	≥61: 20 (40%)	≥61: 14 (28%)
BMI category	Healthy weight: 11 (22%)	Healthy weight: 7 (14%)
	Overweight: 23 (46%)	Overweight: 31 (62%)
	Obese (≥30): 16 (32%)	Obese (≥30): 12 (24%)
Smoking status	Smokers: 17 (34%)	Smokers: 16 (32%)
	Non-smokers: 33 (66%)	Non-smokers: 34 (68%)
Diabetes	27 (54%)	21 (42%)
Impaired fasting glucose	10 (20%)	8 (16%)
Hypertension	17 (34%)	20 (40%)

Laboratory investigations**

Laboratory investigations revealed that only ALT (serum glutamic-pyruvic transaminase (SGPT)) and serum triglyceride levels showed a statistically significant difference between NAFLD cases and controls (p<0.05), while all other parameters showed no significant variation (Table 2).

**Table 2:** Laboratory investigations in NAFLD cases and controls Comparison of biochemical and hematological parameters between NAFLD cases and controls. Data are presented as mean±standard deviation (SD). Statistical analyses were performed using the independent samples t-test. Values with p<0.05 are considered statistically significant. FBS: fasting blood sugar; PPBS: postprandial blood sugar; SGPT: serum glutamic-pyruvic transaminase; ALT: alanine transaminase; SGOT: serum glutamic-oxaloacetic transaminase; AST: aspartate aminotransferase; HDL: high-density lipoprotein; LDL: low-density lipoprotein; VLDL: very-low-density lipoprotein

Investigations	NAFLD cases (mean±SD)	Controls (mean±SD)	Test value (t)	P-value
FBS (mg/dl)	105.35±19.48	99.19±20.21	0.436	0.665
PPBS (mg/dl)	232.22±106.16	194.64±91.22	1.900	0.061
Direct bilirubin (mg/dl)	0.51±0.29	0.47±0.21	0.855	0.397
Indirect bilirubin (mg/dl)	0.32±0.20	0.286±0.15	1.145	0.398
Albumin (g/dL)	3.81±0.75	3.88±0.72	1.356	0.635
SGPT/ALT (IU/L)	42.28±17.91	29.68±15.07	3.41	0.002
SGOT/AST (IU/L)	29.88±24.6	27.48±17.91	0.857	0.578
HDL (mg/dl)	33.42±13.19	34.06±16.89	1.299	0.833
LDL (mg/dl)	82.14±40.37	99.62±56.39	1.390	0.077
VLDL (mg/dl)	31.98±17.77	33.2±21.60	0.890	0.758
Cholesterol (mg/dl)	151.98±46.62	160.0±73.48	1.234	0.516
Triglyceride (mg/dl)	229.8±70.38	158.48± 59.94	3.03	<0.01

Carotid artery intimal thickness

The CIMT was assessed on both the left and right sides in males and females of both the NAFLD and control groups. CIMT was significantly higher in both males and females in NAFLD compared to controls on both left and right sides (p<0.001) (Table 3).

**Table 3:** CIMT in NAFLD cases and controls as per gender Comparison of mean CIMT between NAFLD and control groups stratified by sex. Values are expressed as mean±standard deviation (mm). Statistical analyses were performed using the independent samples t-test. A p-value of <0.05 was considered statistically significant. CIMT: carotid intima-media thickness

Gender	Mean CIMT (mm) left				Mean CIMT (mm) right			
	NAFLD cases	Controls	Test value (t)	P-value	NAFLD cases	Controls	Test value (t)	P-value
Male	0.79±0.15	0.56±0.15	1.382	<0.001	0.82±0.19	0.55±0.15	5.890	<0.001
Female	0.70±0.17	0.49±0.08	3.75	<0.001	0.794±0.19	0.52±0.12	2.89	<0.001

In male participants, the mean left CIMT was 0.79±0.15 mm in the NAFLD group, compared to 0.56±0.15 mm in the control group. The mean right CIMT was 0.82±0.19 mm in NAFLD cases versus 0.55±0.15 mm in controls. Similarly, among female participants, the mean left CIMT was 0.70±0.17 mm in NAFLD cases, compared to 0.49±0.08 mm in controls, while the mean right CIMT was 0.794±0.19 mm in NAFLD cases versus 0.52±0.12 mm in controls.

The study indicates that the left-side CIMT was significantly higher in the NAFLD group compared to the control group across all age groups. Similarly, the right-side CIMT was significantly elevated in the NAFLD group in all age categories, except for the 30-40-year age group, where the difference did not reach statistical significance (Table 4). 

**Table 4:** CIMT in NAFLD cases and controls as per age Comparison of mean CIMT between NAFLD cases and controls across different age groups. Values are presented as mean±standard deviation (mm). Statistical analyses were performed using the independent samples t-test. A p-value of <0.05 was considered statistically significant. "a" indicates that comparative data or statistical testing was not applicable due to missing control group values. CIMT: carotid intima-media thickness; NAFLD: non-alcoholic fatty liver disease

Age group (years)	Mean CIMT (mm) left				Mean CIMT (mm) right			
	NAFLD cases	Controls	Test value (t)	P-value	NAFLD cases	Controls	Test value (t)	P-value
<30	0.7±0.0	-	a	a	0.8±0.0	-	a	a
30-40	0.78±0.10	0.52±0.15	4.56	0.007	0.79±0.20	0.6±0.10	0.77	0.087
41-50	0.72±0.13	0.52±0.071	4.27	0.019	0.86±0.17	0.57±0.14	2.78	0.002
51-60	0.78±0.15	0.51±0.10	4.74	<0.001	0.75±0.22	0.49±0.10	5.80	<0.001
61-70	0.80±0.18	0.55±0.12	3.67	0.007	0.88±0.16	0.59±0.15	3.58	0.002
>70	0.61±0.16	0.56±0.13	3.46	<0.001	0.75±0.15	0.52±0.11	4.89	0.005

Ultrasound findings in NAFLD

**Figure 1:**
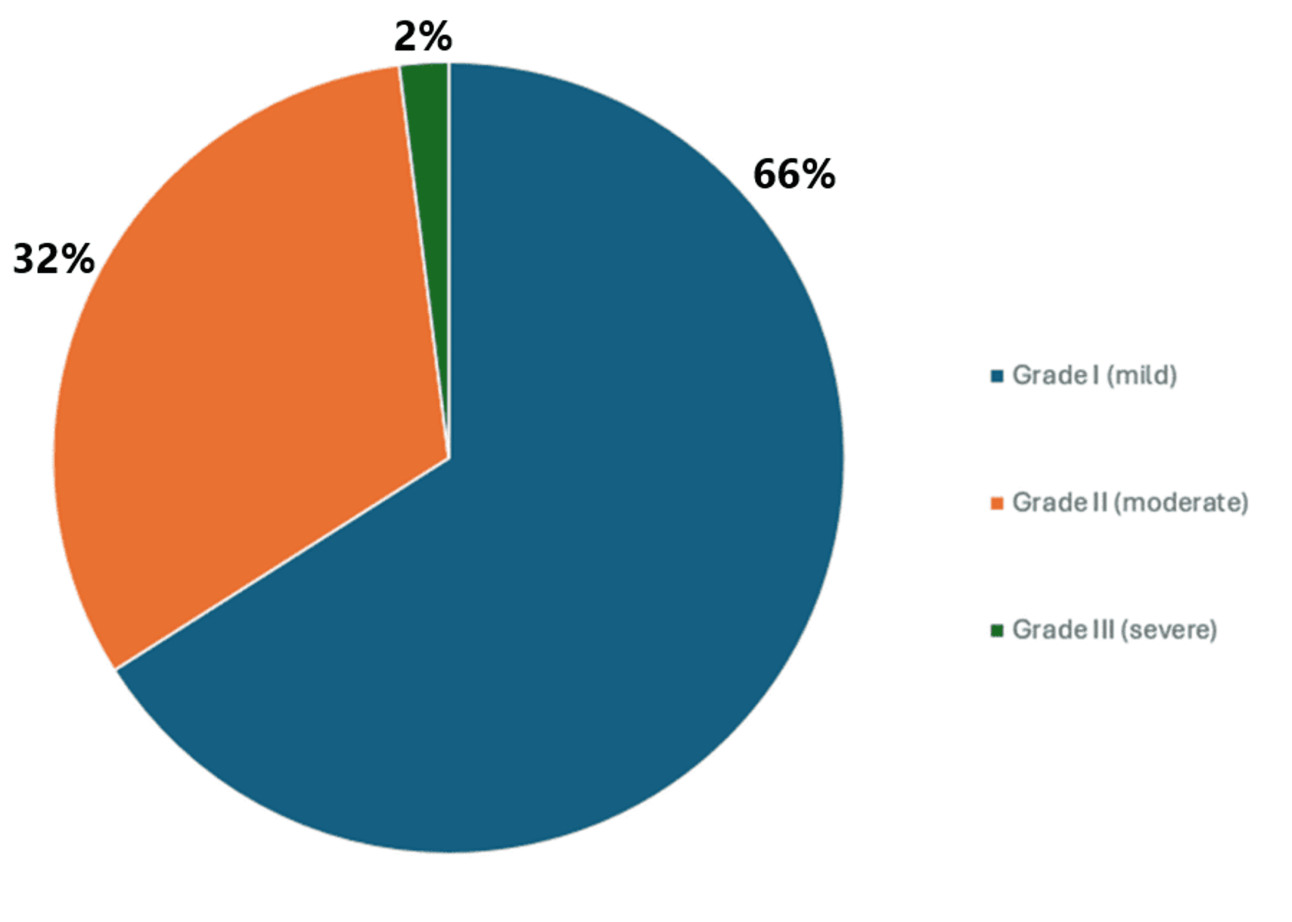
Ultrasonographic staging in the NAFLD group NAFLD: non-alcoholic fatty liver disease

On ultrasound, most of the NAFLD patients had Grade I fatty liver (33 cases; 66%), followed by Grade II (16 cases; 32%), while only one patient (2%) had Grade III, indicating severe steatosis. This shows that the majority of cases were in the mild to moderate stage (Figure 1). 

Correlation between CIMT and serum transaminases

The data indicate a weak but statistically significant inverse correlation between ALT levels and both mean CIMT (r=-0.21; p=0.039) and left CIMT (r=-0.24; p=0.014), suggesting that higher ALT levels are modestly associated with lower CIMT, particularly on the left side. However, no significant correlation was observed between ALT and right CIMT, nor between AST and any of the CIMT parameters (mean, left, or right), indicating that AST does not appear to have a meaningful association with subclinical atherosclerosis in this context (Table 5).

**Table 5:** Correlation between serum transaminases (ALT and AST) and CIMT Correlation between serum transaminases (ALT and AST) and CIMT. Data are expressed as Pearson's correlation coefficient (r) with corresponding p-values. A p-value of <0.05 was considered statistically significant. CIMT: carotid intima-media thickness; ALT: alanine aminotransferase; AST: aspartate aminotransferase

Outcome variable	Predictor variable			
	Serum transaminases			
CIMT	ALT		AST	
	Correlation coefficient "r"	P-value	Correlation coefficient "r"	P-value
CIMT mean	-0.21	0.039	-0.099	0.327
CIMT left	-0.24	0.014	-0.091	0.369
CIMT right	-0.127	0.209	-0.080	0.427

## Discussion

NAFLD is a condition where fat builds up in the liver without significant alcohol use. It can range from simple fat accumulation (steatosis) to more serious liver damage like inflammation (steatohepatitis), cirrhosis, and even liver failure. NAFLD is often linked with metabolic syndrome factors such as diabetes, abnormal cholesterol levels, obesity, and high blood pressure. In this case-control study, we explored the connection between NAFLD and atherosclerosis by measuring the thickness of the carotid artery walls, known as CIMT. 

Baseline characteristics of the study population

The overall mean age of participants was 57.52 years, with the NAFLD group averaging 57.54 years and the control group 55.42 years. NAFLD was found to be more prevalent among older individuals and was more common in males, accounting for 64% of cases.

These results are consistent with previous studies. Summart et al. reported that 64.5% of female NAFLD patients were over 50 years old, with a mean age of 57.8 years, similar to our study [13]. Similarly, Golabi et al. found that 57.79% of NAFLD patients were male, with a mean age of 51.31 years [14]. Kim et al. also observed a higher prevalence of NAFLD in men, particularly those aged 60 years and above, supporting the current study's findings [15].

In the present study, 23 (46%) NAFLD patients were classified as overweight (BMI 25.0-29.9 kg/m²), and 16 patients (32%) had Grade I obesity (BMI 30.0-34.9 kg/m²), as per the World Health Organization (WHO) classification of BMI for adults [16]. These results underscore the strong association between NAFLD and excess body weight, reinforcing its well-recognized relationship with metabolic syndrome, particularly obesity.

Similar findings were reported in previous studies: Brea et al. observed an average BMI of 31.8±5.1 kg/m², Uslusoy et al. reported an average BMI of 30.6±5.24 kg/m², and Kang et al. noted an average BMI of 25.9±3.07 kg/m² among NAFLD patients [17-19].

In the present study, 54% of NAFLD patients had diabetes and 34% were smokers. Comparable findings have been reported in previous studies. Uslusoy et al. observed diabetes in 20.3% and smoking in 26.5% of NAFLD cases [18]. Kang et al. reported 36.87% smokers but no diabetic patients, while Moon et al. found 38% with diabetes and 32.7% smokers [19,20]. Similarly, Albricker et al. reported diabetes in 47% and smoking in 45% of their NAFLD patients [21].

Laboratory investigations analysis

In the present study, fasting blood sugar (105.35±19.48), AST (29.88±24.6), ALT (42.28±17.91), HDL (33.42±13.19), and low-density lipoprotein (LDL) (82.14±40.37) levels were found to be higher in NAFLD patients compared to the control group. However, triglycerides and ALT showed a statistically significant difference (p<0.05), while the differences in other parameters were not statistically significant, indicating minimal variation between the two groups.

These findings are consistent with studies by Uslusoy et al., Kang et al., and Zhang et al., who reported similar biochemical trends in NAFLD patients [18,19,22]. One exception was the LDL levels in the study by Kang et al., which differed from our findings, possibly due to differences in sample size [19]. 

Lipid fraction analysis

In our findings, LDL levels above 100 mg/dl were seen in 34% of NAFLD cases, which was not significantly different from the control group (p>0.05). The elevated triglycerides (>150 mg/dl) were observed in 84% of the NAFLD cases, showing a statistically significant difference from controls (p<0.05). The rest of the lipid parameters were not significantly different from the control group. 

These findings are consistent with Latif et al., who reported raised levels of triglycerides, cholesterol, LDL, and non-HDL in a majority of NAFLD patients, with low HDL-C in 90.5% of cases [23]. Similarly, Mahaling et al. found significant differences in triglycerides, cholesterol, LDL, and non-HDL, though they did not find a strong link between triglycerides and NAFLD grade [24]. Bano et al. also identified hypertriglyceridemia as the most frequent lipid abnormality, followed by low HDL-C and hypercholesterolemia [25].

CIMT analysis

In the present study, mean CIMT on both the left and right sides was significantly greater in patients with NAFLD compared to controls, in both males and females (p<0.05). This observation is consistent with the findings of Kim et al., who also reported significant sex-specific differences in CIMT [15]. Furthermore, mean CIMT values and the prevalence of carotid plaques were markedly higher among individuals with hypertension or diabetes compared to those without these conditions (p<0.05), corroborating the results of Kang et al. and Targher and Arcaro, who documented a similar increase in CIMT among NAFLD patients relative to controls [19,26].

Notably, in our cohort, the difference in CIMT between NAFLD patients and controls was more pronounced in participants aged ≥50 years, with statistical significance increasing from p<0.05 to p<0.001. This suggests that older adults are more susceptible to CIMT progression and, consequently, to elevated cardiovascular risk. These results parallel those of Chouhan et al. who reported a progressive age-related increase in CIMT within the NAFLD population, with significant left- and right-sided differences compared to controls [27].

Similarly, Jadhav and Kadam noted that CIMT in the general population typically ranges from 0.4 to 1 mm, increasing by 0.01-0.33 mm annually, with a faster rate (0.03-0.06 mm/year) in patients with coronary artery disease (CAD) [28]. This underscores that CIMT progression is age-dependent and accelerates substantially after the age of 50, aligning with the observed sharp rise in cardiovascular event risk in this age group. Collectively, our findings and the supporting literature indicate that age is a critical determinant of CIMT progression, particularly in individuals with NAFLD.

Ultrasound abdomen analysis

In the present study, the majority of NAFLD patients were classified as Grade I (mild) on ultrasound (66.6%), followed by Grade II (moderate) (32%), with only 2% falling into Grade III (severe). These results align closely with the findings of Khadka et al. who reported 55.1% mild, 32.7% moderate, and 12.2% severe cases [29]. Similar patterns were also observed in studies by Razavizade et al. and Fu et al., indicating that most NAFLD cases tend to present at an early, milder stage [30,31].

Correlation between CIMT and NAFLD

This study evaluated the association between CIMT and NAFLD by comparing CIMT measurements in patients with NAFLD and healthy controls. The results demonstrated that the mean CIMT in individuals with fatty liver disease was significantly higher than that of the control group.

This result was aligned with the previous studies. For example, a meta-analysis by Sookoian et al., which included seven studies with 1,427 NAFLD patients and 2,070 healthy individuals, found a strong correlation between NAFLD and increased CIMT, indicating a link to atherosclerosis [32]. Similarly, Targher and Arcaro reported higher CIMT in NAFLD patients compared to healthy subjects [26]. Fracanzani et al. also observed significantly higher mean CIMT values in 125 NAFLD patients compared to 250 controls [33].

Correlation between CIMT and serum transaminases

In this study, a negative correlation was found between serum transaminase levels and CIMT, with the correlation coefficient "r" being negative in all cases. However, this association was not statistically significant (p>0.05), except for the mean CIMT and left mean CIMT in relation to ALT, which showed a significant association (p<0.05).

Shanaki et al. also reported a negative, but insignificant, correlation between CIMT and transaminase levels, suggesting transaminase is a weak predictor of CIMT [34]. Another study by Yoon et al. suggested that adiponectin may be an independent factor linked to atherosclerosis [35]. Overall, it appears that lower transaminase levels might be connected to subclinical atherosclerosis, but larger studies are needed to confirm this. 

Limitations of the study

The study has several limitations that should be considered. The relatively small sample size may limit the generalizability of the findings and reduce the ability to detect subtle associations. Since the research was conducted in a single hospital setting, the results may not reflect the broader population or community-based scenarios. The case-control design captures data at a single point in time, which makes it difficult to establish a causal relationship between NAFLD, elevated ALT levels, and increased CIMT.

While ultrasonography is a practical, non-invasive tool for diagnosing and grading NAFLD, its accuracy is highly dependent on the operator's expertise, and it may lack the sensitivity required to detect early or mild disease when compared with advanced imaging modalities, liver elastography, or liver biopsy. Furthermore, the absence of histological confirmation is a limitation, as liver biopsy remains the gold standard for definitive NAFLD diagnosis.

The study may not have fully accounted for all potential confounding factors such as lifestyle, dietary habits, genetic factors, and inflammatory markers that could influence CIMT or liver enzyme levels. Most patients had mild NAFLD, which restricts the ability to evaluate the effects of more advanced disease on vascular health. Furthermore, the absence of longitudinal follow-up data means the study cannot assess how NAFLD or CIMT may progress over time or respond to interventions.

## Conclusions

This case-control study establishes an association between NAFLD and increased CIMT, suggesting that individuals with NAFLD are at greater risk for subclinical atherosclerosis and CVD. NAFLD was more prevalent in men and those over the age of 50, and it was closely linked with features of metabolic syndrome such as obesity, diabetes, and dyslipidemia. Most patients had mild fatty liver and showed elevated triglycerides, low HDL, and significantly higher ALT levels, indicating that ALT may serve as a more reliable marker of liver involvement than AST.

CIMT was significantly elevated in NAFLD patients, especially in older individuals and those with more severe grades of steatosis on ultrasound. The study highlights the usefulness of imaging in assessing both liver and vascular health. Although CIMT correlated well with ALT and NAFLD severity, its relationship with overall liver enzyme levels was weak. These findings support the idea that NAFLD is a systemic condition with cardiovascular implications, not just a liver disease. Therefore, routine cardiovascular risk assessment, including CIMT evaluation, should be considered for all NAFLD patients, particularly those with metabolic syndrome.
